# Traumatic dural sinus thrombosis causing persistent headache in a child

**DOI:** 10.4103/0974-2700.58658

**Published:** 2010

**Authors:** Bhavana Lakhkar, Bhushan Lakhkar, Brij Raj Singh, Amit Agrawal

**Affiliations:** Datta Meghe Institute of Medical Sciences, Sawangi (Meghe), Wardha, India

**Keywords:** Head injury, lateral sinus, sigmoid sinus, sinus thrombosis

## Abstract

Dural venous sinus thrombosis following a mild head injury is increasingly recognized. We report case of a 9-year-old male child presented with progressive headache and vomiting following a minor fall. A diagnosis of sinus venous thrombosis was suspected on nonenhancing computed tomography, and that was confirmed with magnetic resonance venography. The child was managed with intravenous fluids, anticoagulation (injection heparin followed by oral anticoagulants–tab coumarin), antiedema measures (mannitol), and antiepileptics (phenytoin) with good outcome.

## INTRODUCTION

Intracranial dural venous sinus thrombosis secondary to mild closed head injury without cranial vault fractures or intracranial hematomas is an increasingly recognized entity in the literature.[1–6]

## CASE REPORT

**Figure 1:**
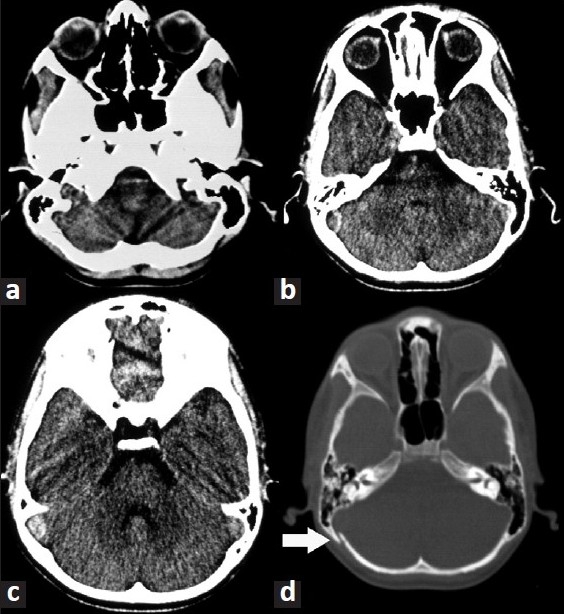
CT scan (a–d) showing hyperdense area in the region of right sigmoid sinus and sutural diastases of right lambdoid suture (arrow in D).

**Figure 2:**
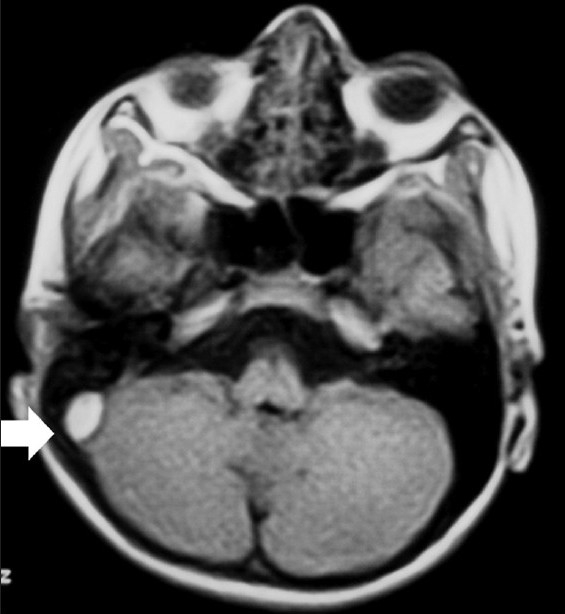
MRI T1W image showing iso- to hyperintense area in the region of right sigmoid sinus (arrow)

**Figure 3:**
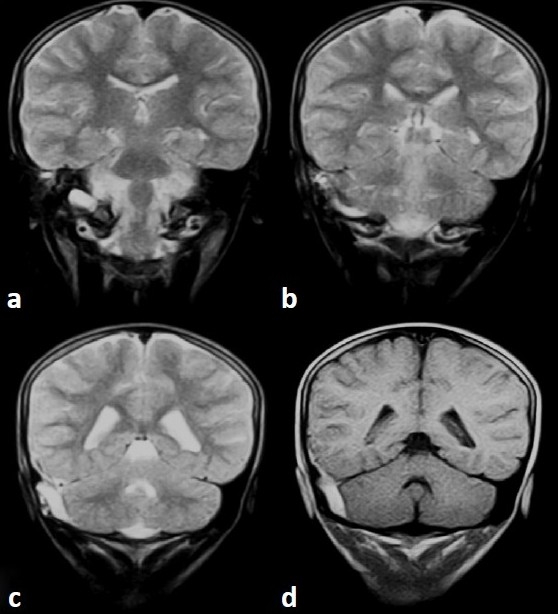
MRI T2W image (a–d) showing hyperintense area in the region of right sigmoid sinus

**Figure 4:**
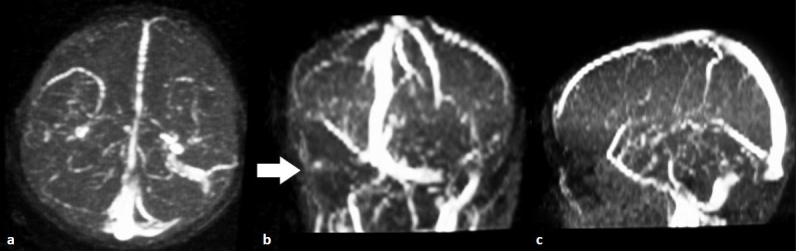
MR venogram (a–c) showing nonvisualization of the right transverse and sigmoid sinus (arrow in B)

A 9-year-old male child presented with the history of fall form stairs (approximately 2 feet height) of 1-day duration. He lost consciousness for 2–3 min and was complaining of mild headache on recovery. Over the next 24 h, he developed worsening in headache and had multiple episodes of vomiting. Although at the time of admission the child was conscious, but he became progressively drowsy in the ward. Glasgow coma scale (GCS) was E3V5M6. Fundus showed bilateral papilloedema. Other cranial nerves were normal. There were no focal motor or sensory deficits. Computed tomography (CT) scan of the brain (performed after 36 h of injury) showed sutural diastases of right lambdoid suture and hyperdensity in the region of right sigmoid sinus [Figure 1]. Based on CT findings, a diagnosis of sigmoid sinus thrombosis was suspected. Further investigations with magnetic resonance imaging (MRI) showed an isointense to hyperintense area on T1W images becoming hyperintense on T2W images in the region of right transverse and sigmoid sinus [Figures 2 and 3]. MR angiography further confirmed the occlusion of the right transverse and sigmoid sinus [Figure 4, arrow in b]. Detailed blood investigations including hemoglobin, platelet count, prothrombin time, bleeding, and clotting time were within normal limits. We did not anticipate the diagnosis of sinus thrombosis clinically; only after radiological investigations, the diagnosis was made. The child was managed with intravenous fluids, anticoagulation (injection heparin followed by oral anticoagulants—tab coumarin), antiedema measures (mannitol), and antiepileptics (phenytoin). As the child was doing well at follow-up, a repeat scan was not performed.

## DISCUSSION

The common causes of intracranial dural venous sinus thrombosis include head and neck infections, pregnancy and puerperium, use of oral contraceptives, and dehydration.[1] Following head injury, skull fractures or intracranial hematomas can cause thrombosis either by direct compression of the sinus[2] or endothelial damage within the sinus can cause the activation of the coagulation system resulting in sinus occlusion.[4] Uncommonly, sinus thrombosis can occur after mild closed head injury with sutural diastasis.[1] Clinical symptoms of sinus thrombosis include features of increased intracranial pressure such as headache, papilloedema, impaired consciousness, and vomiting.[1,2] Proposed mechanisms of sinus thrombosis after mild closed head injury involve endothelial damage within the venous sinus that may cause thrombosis by activating the coagulation system.[3] The brain also contains an abundance of thromboplastin that is released after injury inducing an abnormal hypercoagulable state leading to the destruction of platelets and erythrocytes followed by thrombus formation.[1] The characteristic CT findings of sinus thrombosis include the cord sign, dense vein sign, and empty delta sign following the administration of contrast agents.[1–4] “Empty triangle” has been described as a reliable finding on CT scan and it is due to the presence of an isodense clot within the sinus enclosed by an area of engorged vessels.[1,3,5] Digital substraction angiography is the method of choice for diagnosing sinus thrombosis. The complete absence of the transverse and sigmoid sinus may be normal anatomic variation and thrombosis involving these areas should be made cautiously.[1–4] In the present case, the MRI findings were extremely valuable to establish the diagnosis during the acute phase of thrombus formation, as it showed the thrombus as well as signal intensities characteristic of oxyhemoglobin. Magnetic resonance venography (MRV) is superior to CT but cerebral angiography, though invasive is the gold standard for the diagnosis of CVT. MRV is particularly helpful in children as it does not require contrast medium and can approximately tell the age of thrombus, and the sequential follow-up is also possible.[1,2,4] The lumbar puncture can be performed if there is suspicion of benign intracranial hypertension.[3] The treatment of sinus thrombosis is controversial and includes the stabilization of the patient in acute phase (hydration, anticonvulsants, steroids, mannitol, acetazolamide, and craniectomy for decreasing intracranial pressure) and investigations for any known risk factors including prevention and management of deep venous thrombosis, infection, and coagulopathy.[2,3,5–8] In the present case, favorable outcome can be attributed to the fact that fibrinolytic activity is higher in normal venous walls than in artery or capillary walls, and thrombi in the sinuses frequently recanalize with time due to fibrinolysis.[1]

## CONCLUSION

Although with limited resources it was not possible to perform all recommended investigations in the present case to rule out other cases of sinus thrombosis, circumstantial evidence supported the diagnosis of post-head-injury sinus thrombosis (as there was no history of any previous major illness in this child, i.e., infection, dehydration, bleeding disorders, or any thrombotic episodes). In summary, although sinus thrombosis following minor head injury is increasingly recognized in children and it should be considered as a differential diagnosis when there is persistent headache after head injury. Early detection is important as early management with anticoagulation of this potentially treatable condition will result in good outcome.
